# Supplementary material for: Endothelial ZEB1 promotes angiogenesis-dependent bone formation and reverses osteoporosis
Source: Nat Commun. 2020 Jan 23;11:460. doi: 10.1038/s41467-019-14076-3 (PMC6978338; doi:10.1038/s41467-019-14076-3)
Supplement: Supplementary file 1 — Reporting Summary [file 41467_2019_14076_MOESM1_ESM.pdf]

natureresearch

Corresponding author(s): Zhao-Qiu Wu

Last updated by author(s): Dec. 4, 2019

Reporting Summary

Nature Research wishes to improve the reproducibility of the work that we publish. This form provides structure for consistency and transparency in reporting. For further information on Nature Research policies, see Authors & Referees and the Editorial Policy Checklist .

Statistics

For all statistical analyses, confirm that the following items are present in the figure legend, table legend, main text, or Methods section.

n/a	Confirmed	
☐	☒	The exact sample size (*n*) for each experimental group/condition, given as a discrete number and unit of measurement
☐	☒	A statement on whether measurements were taken from distinct samples or whether the same sample was measured repeatedly
☐	☒	The statistical test(s) used AND whether they are one- or two-sided<br>*Only common tests should be described solely by name; describe more complex techniques in the Methods section.*
☒	☐	A description of all covariates tested
☒	☐	A description of any assumptions or corrections, such as tests of normality and adjustment for multiple comparisons
☐	☒	A full description of the statistical parameters including central tendency (e.g. means) or other basic estimates (e.g. regression coefficient) AND variation (e.g. standard deviation) or associated estimates of uncertainty (e.g. confidence intervals)
☒	☐	For null hypothesis testing, the test statistic (e.g. *F*, *t*, *r*) with confidence intervals, effect sizes, degrees of freedom and *P* value noted<br>*Give P values as exact values whenever suitable.*
☒	☐	For Bayesian analysis, information on the choice of priors and Markov chain Monte Carlo settings
☒	☐	For hierarchical and complex designs, identification of the appropriate level for tests and full reporting of outcomes
☒	☐	Estimates of effect sizes (e.g. Cohen's *d*, Pearson's *r*), indicating how they were calculated

Our web collection on statistics for biologists contains articles on many of the points above.

Software and code

Policy information about availability of computer code

Data collection	Immunofluorescent stainings were analysed with Image J software.<br>Mirco-CT data were collected with the data analysis and three-dimensional model visualization software (Hiscan Analyzer V3.0).<br>For flow cytometry, data were collected on a BD FACS Verse flow cytometer.<br>For luciferase reporter assay, data were collected on Thermo Varioskan Flash Multimode Reader.<br>Q-PCR was performed using an ABI 7300 sequence detection system.
Data analysis	GraphPad Prism software.

For manuscripts utilizing custom algorithms or software that are central to the research but not yet described in published literature, software must be made available to editors/reviewers. We strongly encourage code deposition in a community repository (e.g. GitHub). See the Nature Research guidelines for submitting code & software for further information.

Data

Policy information about availability of data

All manuscripts must include a data availability statement. This statement should provide the following information, where applicable:

- Accession codes, unique identifiers, or web links for publicly available datasets
- A list of figures that have associated raw data
- A description of any restrictions on data availability

The source data underlying Figs. 1b, 1d, 1e, 1g, 1i, 2c, 2d, 2e, 2g, 2h, 2k, 2n, 2p, 2r, 2t, 2v, 3a, 3d, 3e, 3g, 4b, 4c, 4e, 4f, 4i, 5d, 5f, 5h, 6b, 6d, 6f, 6h, 7b, 7d and Supplementary Figs. 1c, 1e, 1g, 2b, 2d, 2f, 2h, 3a, 3c, 3f, 3h, 3j, 4b, 4d, 4f, 4h, 5a, 5c, 5d, 6b, 6c, 6d, 6f, 7b are provided as a Source Data file. Unprocessed original scans of blots are shown in Supplementary Fig. 8. The remaining data is contained within the Article, Supplementary Information or available from the authors upon request. A reporting summary for this Article is available as a Supplementary Information file.

Field-specific reporting

Please select the one below that is the best fit for your research. If you are not sure, read the appropriate sections before making your selection.

- [x] Life sciences
- [ ] Behavioural & social sciences
- [ ] Ecological, evolutionary & environmental sciences

For a reference copy of the document with all sections, see nature.com/documents/nr-reporting-summary-flat.pdf

Life sciences study design

All studies must disclose on these points even when the disclosure is negative.

Sample size	There were at least 3 samples (mice) in independent experiments.
Data exclusions	No animals or samples were excluded from analysis.
Replication	Experiments were repeated at least 3 times to ensure reproducibility except where otherwise indicated in the figure legends.
Randomization	No method of randomization was used as mice were segregated into groups based on genotype and surgical operations.
Blinding	The investigators were not blinded to allocation during experiments and outcome assessment.

Reporting for specific materials, systems and methods

We require information from authors about some types of materials, experimental systems and methods used in many studies. Here, indicate whether each material, system or method listed is relevant to your study. If you are not sure if a list item applies to your research, read the appropriate section before selecting a response.

Materials & experimental systems

n/a	Involved in the study	
☐	☒	Antibodies
☐	☒	Eukaryotic cell lines
☒	☐	Palaeontology
☐	☒	Animals and other organisms
☐	☒	Human research participants
☒	☐	Clinical data

Methods

n/a	Involved in the study	
☒	☐	ChIP-seq
☐	☒	Flow cytometry
☒	☐	MRI-based neuroimaging

Antibodies

Antibodies used	Detailed antibody information including vendor, catalog number and clone name was described in the Methods.
Validation	All antibodies were validated by the manufacturer.

Eukaryotic cell lines

Policy information about cell lines

Cell line source(s)	Primary cells were isolated and used in our laboratory, and HEK293T cell line was purchased from ATCC.
Authentication	None of the cell lines used were authenticated.
Mycoplasma contamination	Cells were tested for mycoplasma contamination every 2 months, and only mycoplasma-negative cells were used.
Commonly misidentified lines (See ICLAC register)	No commonly misidentified cell lines were used for this study.

Animals and other organisms

Policy information about studies involving animals; ARRIVE guidelines recommended for reporting animal research

Laboratory animals	Mice were housed under standard specific-pathogen-free (SPF) conditions and all animal experiments were performed in accordance with protocols approved by the Animal Welfare and Ethics Committee of China Pharmaceutical University (AWEC-CPU). Mice carrying ZEB1 (exon 3) floxed alleles (ZEB1fl/fl) were generated in our laboratory and have been described

previously15. Tie2-Cre transgenic mice were purchased from Jackson Laboratory (#008863). Cdh5(PAC)-CreERT2 transgenic mice were kindly provided by Ralf H. Adams (Max Planck Institute for Molecular Biomedicine, Münster, Germany; ref. 18). For generation of intrinsic and inducible EC-specific ZEB1-deleted mice, ZEB1fl/fl mice were bred with Tie2-Cre and Cdh5(PAC)-CreERT2 transgenic mice, respectively. To induce Cdh5(PAC)-CreERT2 activity and gene inactivation in pups, mice at P8 were i.p. injected with 0.1 mg tamoxifen (10 µl, 10 mg/ml, dissolved in 1:10 ethanol/corn oil; Sigma-Aldrich, #T5648) every day for 7 consecutive days. To induce gene inactivation in adults, 7-week-old mice were i.p. injected with 1.0 mg tamoxifen (100 µl, 10 mg/ml) every other day for 2 consecutive weeks. For r.Dll4 administration experiments, ZEB1fl/fl;Tie2-Cre- (ZEB1WT) and ZEB1fl/fl;Tie2-Cre+ (ZEB1ΔEC) mice at P4 were i.p. injected with r.Dll4 protein (R & D Systems, #1389-D4-050) at a dose of 1 µg/g for 2 consecutive weeks before analysis at P21. To generate OVX and Sham mouse models, the surgery was performed on 8-week-old female mice, and the mice at day 3 post-surgery were i.v. injected with 4 µg Lipo.-Vector-GFP or Lipo.-ZEB1-GFP twice every week for consecutive 6 weeks. Mice were then sacrificed and uterine, long bone, and non-skeletal organs were dissected for further use. All mice were kept in C57BL/6J background and gender-matched littermate controls were used in all experiments. The investigators were not blinded to allocation during experiments and outcome assessment. No method of randomization was used as mice were segregated into groups based on genotype alone. No statistical method was used to predetermine sample size.

Wild animals

The study did not involve wild animals.

Field-collected samples

The study did not involve samples collected from the field.

Ethics oversight

All animal experiments were performed in accordance with protocols approved by the Animal Welfare and Ethics Committee of China Pharmaceutical University (AWEC-CPU).

Note that full information on the approval of the study protocol must also be provided in the manuscript.

Human research participants

Policy information about studies involving human research participants

Population characteristics

Human bone samples were obtained from patients (female) based on the inclusion and exclusion criteria. Six patients with osteoporosis undergoing knee joint replacement with ages ranging from 58 to 75 years and six patients with tibia fracture undergoing Open Reduction Internal Fixation ranging in age from 12 to 30 years (human bone sample collection was conducted at the Division of Orthopedic Surgery of The Affiliated Nanjing Hospital, Nanjing Medical University, Nanjing, China).

Recruitment

All subjects were screened using a detailed questionnaire, disease history and physical examination. These participants were performed bone sample collection during knee joint replacement and bone fracture surgery.

Ethics oversight

The clinical study was approved by the Ethnic Committee of The Affiliated Nanjing Hospital, Nanjing Medical University. Written informed consents were obtained from each participant before procedure.

Note that full information on the approval of the study protocol must also be provided in the manuscript.

Flow Cytometry

Plots

Confirm that:

- [x] The axis labels state the marker and fluorochrome used (e.g. CD4-FITC).
- [x] The axis scales are clearly visible. Include numbers along axes only for bottom left plot of group (a 'group' is an analysis of identical markers).
- [x] All plots are contour plots with outliers or pseudocolor plots.
- [x] A numerical value for number of cells or percentage (with statistics) is provided.

Methodology

Sample preparation

Tibias were dissected from mice. Metaphysis and diaphysis regions of the bone were crushed in ice-cold PBS after removal of epiphysis and muscles. Whole bone marrow was digested with collagenase incubation at 37 °C for 25 min to obtain a single cell suspension. After filtration and washing, equal number of cells were immunostained with Endomucin antibody (SC-65495, Santa Cruz) for 45 min at 4°C. After washing, cells were stained with PE-CD45 (553081, BD Pharmingen), PE-Ter119 (553673, BD Pharmingen), PE-cy7-CD31 (561410, BD Pharmingen) and goat anti-rat antibody coupled to AF647 (A-21248, Invitrogen) for 45 min at 4°C, followed by DAPI staining for 5 min before analysis and sorting.

Instrument

Cells were acquired on a BD FACS Verse flow cytometer and sorted on a FACS Aria II flow cytometer.

Software

FlowJo 10 was used to analyze the data.

Cell population abundance

We sorted CD31+CD45-Ter119- cells as total bone endothelial cells, CD31hiEMCNhiCD45-Ter119- cells as type H bone endothelial cells, and CD31loEMCNloCD45-Ter119- cells as type L bone endothelial cells

Gating strategy

To demarcate and sort CD31hiEMCNhi cells, first standard quadrant gates were set. To differentiate CD31hiEMCNhi cells from the total double positive cells in quadrant 2 gates were set at > 104 log Fl-2 (PE-Cy7-CD31) fluorescence and > 104 log Fl-4 (Alexa

Fluor 647-Endomucin) fluorescence. DAPI-CD45-Ter119-CD31+ cells were sorted according to side scatter and set at > 103 log Fl-2 fluorescence (PE-Cy7-CD31) after negative selection of CD45 and Ter119 at < 103 log Fl-2 (PE-CD45 and APC-Ter119) fluorescence. DAPI-CD45-Ter119-CD31+ cells were sorted as total bone ECs.

☒ Tick this box to confirm that a figure exemplifying the gating strategy is provided in the Supplementary Information.
