# Supplementary material for: Endothelial ZEB1 promotes angiogenesis-dependent bone formation and reverses osteoporosis
Source: Nat Commun. 2020 Jan 23;11:460. doi: 10.1038/s41467-019-14076-3 (PMC6978338; doi:10.1038/s41467-019-14076-3)
Supplement: Supplementary file 2 — Supplementary Information [file 41467_2019_14076_MOESM2_ESM.pdf]

Supplementary Information

Endothelial ZEB1 promotes angiogenesis-dependent bone formation and reverses osteoporosis

Fu et al

Supplementary Figure 1

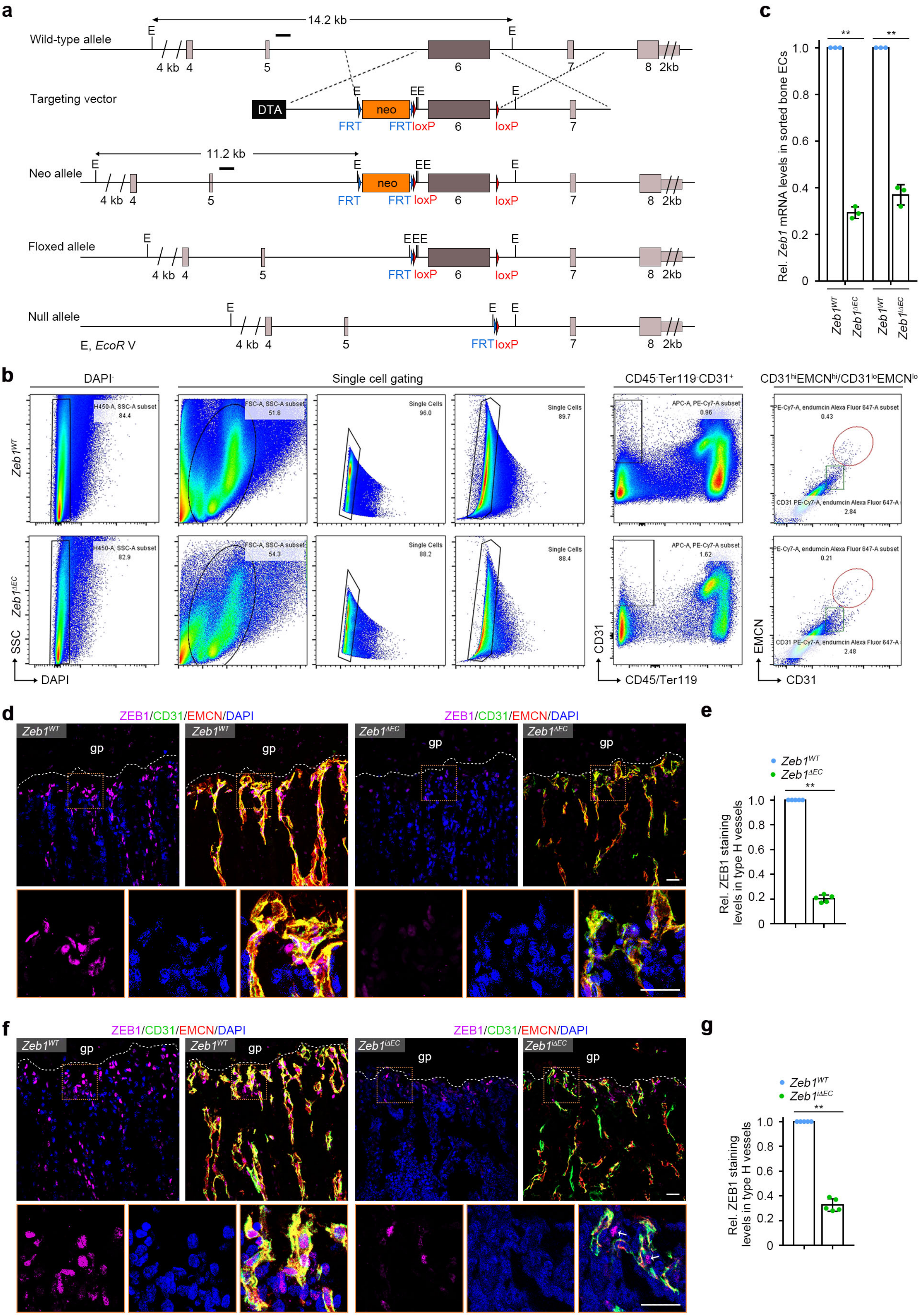

Supplementary Figure 1. ZEB1 is efficiently and selectively excised in bone endothelium of ZEB1 conditional knockout mice. a Schematic representation of the *Zeb1* targeted allele. The genomic structure after neo-cassette removal is shown on the third line. The genomic structure after loxP-cassette removal is shown on the fourth line. DTA, diphtheria toxin cassette; red triangle, loxP sites; purple triangle, FRT sites; neo, neomycin resistance cassette. **b** Schematic representation of the strategy used for FACS-sorting of $DAPI^-CD45^-Ter119^-CD31^+$ bone ECs, $DAPI^-CD45^-Ter119^-CD31^{hi}EMCN^{hi}$ type H bone ECs, and $DAPI^-CD45^-Ter119^-CD31^{lo}EMCN^{lo}$ type L bone ECs from 3-week-old $Zeb1^{WT}$ and $Zeb1^{\Delta EC}$ mice. **c** RT-qPCR analysis of *Zeb1* transcript in FACS-sorted bone ECs of 3-week-old $Zeb1^{\Delta EC}$ mice (left 2 columns) and $Zeb1^{i\Delta EC}$ mice (right 2 columns) and their corresponding littermate controls (n = 3 independent experiments). **d** Representative confocal images of ZEB1/CD31/EMCN immunostaining in tibia of 3-week-old $Zeb1^{WT}$ and $Zeb1^{\Delta EC}$ mice (n = 5, each). Nuclei, DAPI. Magnified areas of dashed boxed sections are shown in bottom panels. gp, growth plate. Scale bar, 30 μm. **e** Quantification of ZEB1 staining levels in type H vessels of tibia as shown in **d** (n = 5 independent experiments). **f** Representative confocal images of ZEB1/CD31/EMCN immunostaining in tibia of 3-week-old $Zeb1^{WT}$ and $Zeb1^{i\Delta EC}$ mice that were i.p. injected with 0.1 mg tamoxifen every day for 7 consecutive days at 8 days of ages (n = 5, each). Magnified areas of dashed boxed sections are shown in bottom panels. Scale bar, 30 μm. **g** Quantification of ZEB1 staining levels in type H vessels of tibia as shown in **f** (n = 5 independent experiments). All data are represented as mean ± s.d. ** $P < 0.01$. Differences are tested using unpaired two-tailed Student's *t*-test (**c**, **e**, **g**). The source data are provided as a Source Data file.

Supplementary Figure 2

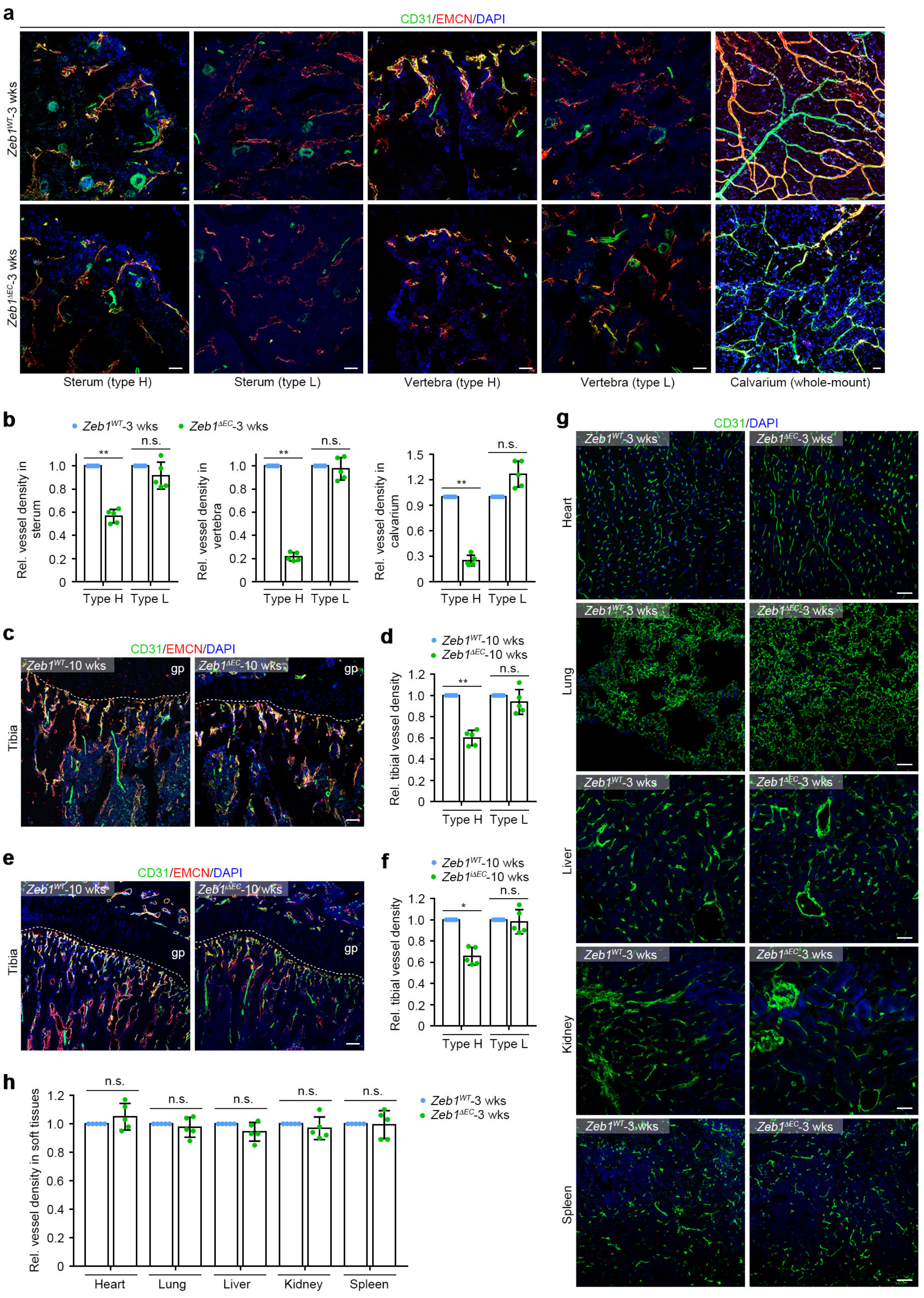

Supplementary Figure 2. Endothelial ZEB1 deletion impairs vessel formation in bone but not in non-skeletal organs. a Representative confocal images of CD31/EMCN immunostaining in skeletal elements (e.g. sternum, vertebra, and calvarium) of 3-week-old *Zeb1*^{WT} and *Zeb1*$^{\Delta EC}$ mice (n = 5, each). Scale bar, 30 μm. **b** Quantification of type H and type L vessel densities in skeletal elements as shown in **a** (n = 5 independent experiments). **c** Representative confocal images of CD31/EMCN immunostaining in tibia of 10-week-old *Zeb1*^{WT} and *Zeb1*$^{\Delta EC}$ mice (n = 5, each). Scale bar, 30 μm. **d** Quantification of type H and type L vessel densities in tibia as shown in **c** (n = 5 independent experiments). **e** Representative confocal images of CD31/EMCN immunostaining in tibia of 10-week-old *Zeb1*^{WT} and *Zeb1*$^{i\Delta EC}$ mice that were i.p. injected with 1.0 mg tamoxifen every other day for 2 consecutive weeks at 7 weeks of ages (n = 5, each). Scale bar, 30 μm. **f** Quantification of type H and type L vessel densities in tibia as shown in **e** (n = 5 independent experiments). **g** Representative confocal images of CD31 immunostaining in non-skeletal organs (e.g. heart, lung, liver, kidney and spleen) of 3-week-old *Zeb1*^{WT} and *Zeb1*$^{\Delta EC}$ mice (n = 5, each). Scale bar, 30 μm. **h** Quantification of vessel densities in non-skeletal organs as shown in **g** (n = 5 independent experiments). All data are represented as mean ± s.d. ** $P < 0.01$, * $P < 0.05$; n.s., not significant. Differences are tested using unpaired two-tailed Student's *t*-test (**b**, **d**, **f**, **h**). The source data are provided as a Source Data file.

Supplementary Figure 3

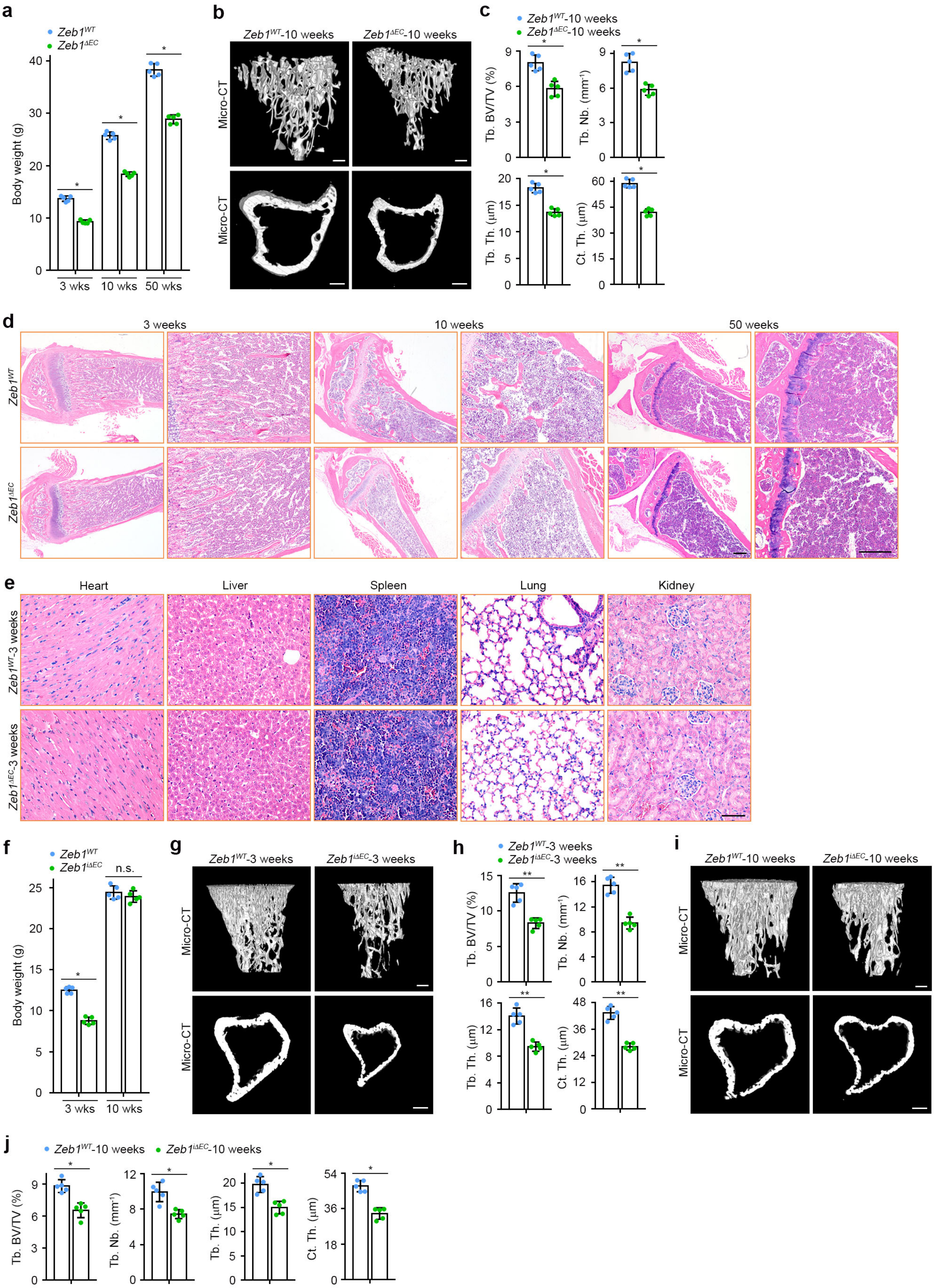

Supplementary Figure 3. Endothelial ZEB1 deletion causes severe defects in bone formation without affecting histological morphology of non-skeletal organs. a Comparisons of body weights of *Zeb1*^{WT} versus *Zeb1*$^{\Delta EC}$ mice at 3, 10, and 50 weeks of ages (n = 5, each). **b** Representative micro-CT images of trabecular bone (top panels) and cortical bone (bottom panels) of 10-week-old *Zeb1*^{WT} and *Zeb1*$^{\Delta EC}$ mice (n = 5, each). Scale bar, 0.2 mm. **c** Quantification of Tb. BV/TV, Tb. Nb., Tb. Th., and Ct. Th. in tibia as shown in **b** (n = 5 independent experiments). **d** Representative images of H&E-stained tibia in *Zeb1*^{WT} and *Zeb1*$^{\Delta EC}$ mice at 3, 10, and 50 weeks of ages (n = 5, each). Magnified images are shown in bottom panels. Scale bar, 100 μm. **e** Representative images of H&E-stained non-skeletal organs (e.g. heart, liver, spleen, lung, and kidney) in 3-week-old *Zeb1*^{WT} and *Zeb1*$^{\Delta EC}$ mice (n = 5, each). Scale bar, 50 μm. **f** Comparisons of body weights between *Zeb1*^{WT} and *Zeb1*$^{i\Delta EC}$ mice at 3 and 10 weeks of ages (n = 5, each). **g** Representative micro-CT images of trabecular bone (top panels) and cortical bone (bottom panels) of 3-week-old *Zeb1*^{WT} and *Zeb1*$^{i\Delta EC}$ mice (n = 5, each). Scale bar, 0.2 mm. **h** Quantification of Tb. BV/TV, Tb. Nb., Tb. Th., and Ct. Th. in tibia as shown in **g** (n = 5 independent experiments). **i** Representative micro-CT images of trabecular bone (top panels) and cortical bone (bottom panels) of 10-week-old *Zeb1*^{WT} and *Zeb1*$^{i\Delta EC}$ mice (n = 5, each). Scale bar, 0.2 mm. **j** Quantification of Tb. BV/TV, Tb. Nb., Tb. Th., and Ct. Th. in tibia as shown in **i** (n = 5 independent experiments). All data are represented as mean ± s.d. ** $P < 0.01$, * $P < 0.05$; n.s., not significant. Differences are tested using unpaired two-tailed Student's *t*-test (**a**, **c**, **f**, **h**, **j**). The source data are provided as a Source Data file.

Supplementary Figure 4

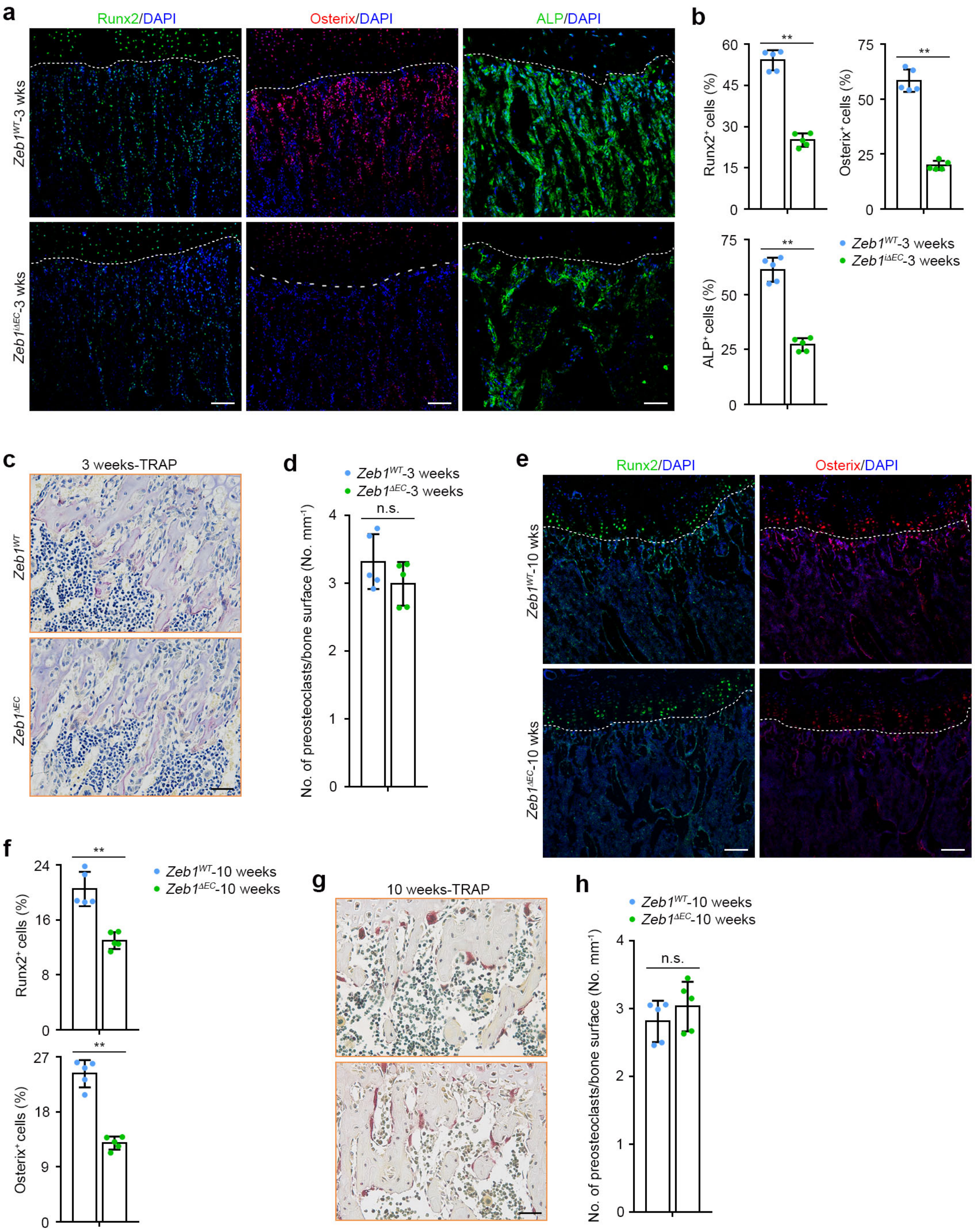

Supplementary Figure 4. Endothelial ZEB1 deletion reduces osteogenesis. a Representative images of 3-week-old *Zeb1*^{WT} and *Zeb1*$^{i\Delta EC}$ tibia (n = 5, each) immunostained with Runx2, Osterix, and ALP. Scale bar, 100 μm. **b** Quantification of Runx2^{+}, Osterix^{+}, and ALP^{+} cells in tibia as shown in **a** (n = 5 independent experiments). **c** Representative images for TRAP staining of 3-week-old *Zeb1*^{WT} and *Zeb1*$^{i\Delta EC}$ tibia (n = 5, each). Scale bar, 100μm. **d** Quantification of TRAP^{+} pre-osteoclast numbers (per bone surface) in tibia as shown in **c** (n = 5 independent experiments). **e** Representative images of 10-week-old *Zeb1*^{WT} and *Zeb1*$^{i\Delta EC}$ tibia (n = 5, each) immunostained with Runx2 and Osterix. Scale bar, 100 μm. **f** Quantification of Runx2^{+} and Osterix^{+} cells in tibia as shown in **e** (n = 5 independent experiments). **g** Representative images for TRAP staining of 10-week-old *Zeb1*^{WT} and *Zeb1*$^{i\Delta EC}$ tibia (n = 5, each). Scale bar, 100μm. **h** Quantification of TRAP^{+} pre-osteoclast enrichment (i.e. TRAP^{+} cell numbers per bone surface) in tibia as shown in **g** (n = 5 independent experiments). All data are represented as mean ± s.d. ** $P < 0.01$; n.s., not significant. Differences are tested using unpaired two-tailed Student's *t*-test (**b**, **d**, **f**, **h**). The source data are provided as a Source Data file.

Supplementary Figure 5

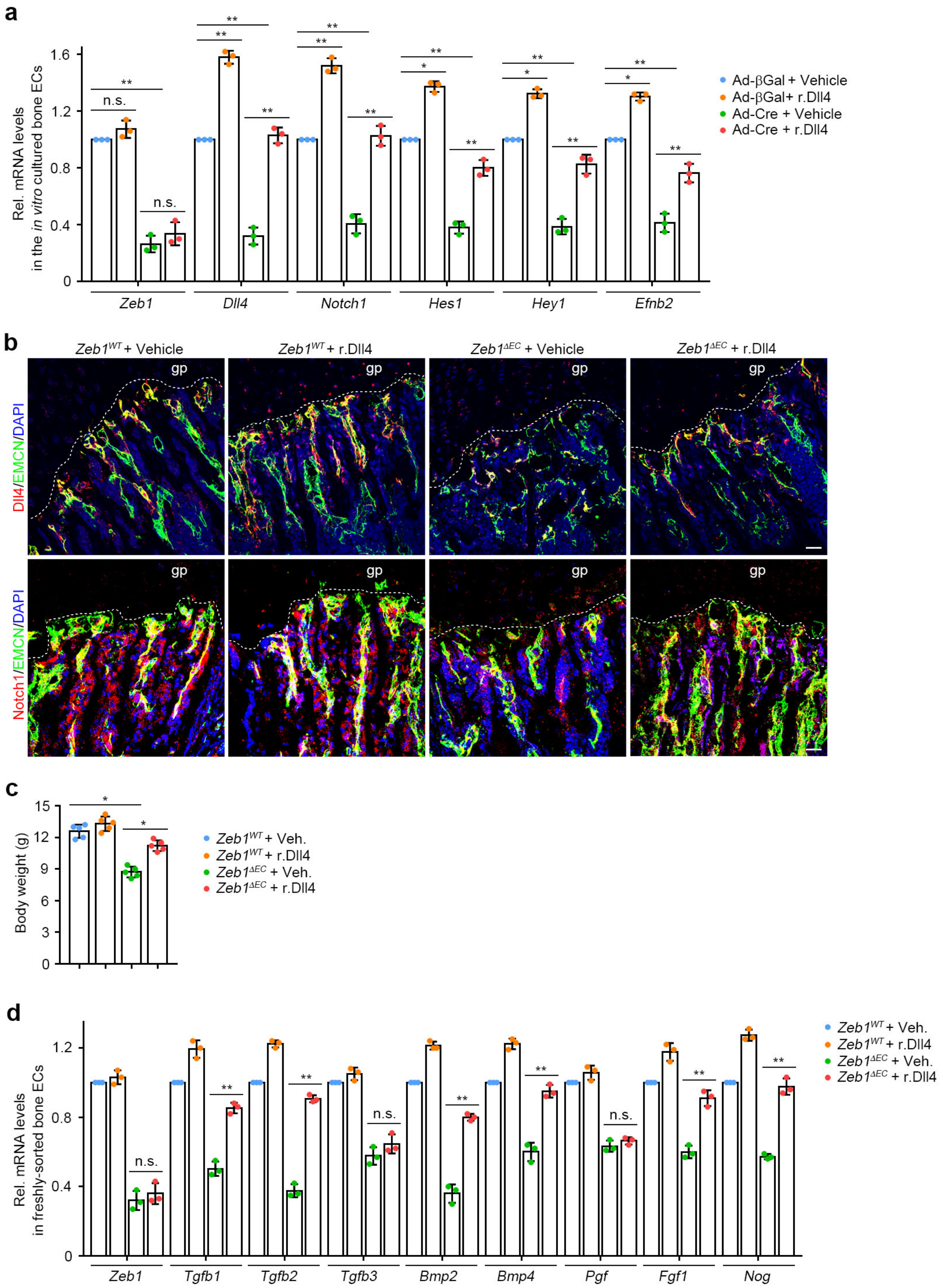

Supplementary Figure 5. Administration of *Zeb1*$^{\Delta EC}$ mice with r.Dll4 protein efficiently restores the impaired Notch activity. a RT-qPCR analysis of *Zeb1*, *Dll4*, *Notch1*, *Hes1*, *Hey1*, and *Efnb2* transcripts in control and ZEB1-deleted bone ECs that were seeded on vehicle- or r.Dll4-precoated culture dishes (n = 3 independent experiments). **b** Representative confocal images of Dll4/EMCN (top panels) and Notch1/EMCN (bottom panels) immunostaining in tibia of *Zeb1*^{WT} and *Zeb1*$^{\Delta EC}$ mice that were i.p. injected with 1 μg/g r.Dll4 protein at P4 for 2 consecutive weeks before analysis at P21 (n = 5, each). Scale bar, 100 μm. **c** Comparisons of body weights of mice as shown in **b** (n = 5, each). **d** RT-qPCR analysis of *Zeb1*, *Tgfb1*, *Tgfb2*, *Tgfb3*, *Bmp2*, *Bmp4*, *Pgf*, *Fgf1* and *Nog* transcripts in FACS-sorted bone ECs of mice as shown in **b** (n = 3 independent experiments). All data are represented as mean ± s.d. ** $P < 0.01$, ** $P < 0.01$; n.s., not significant. Differences are tested using one-way ANOVA with Tukey's post hoc test (**a**, **c**, **d**). The source data are provided as a Source Data file.

Supplementary Figure 6

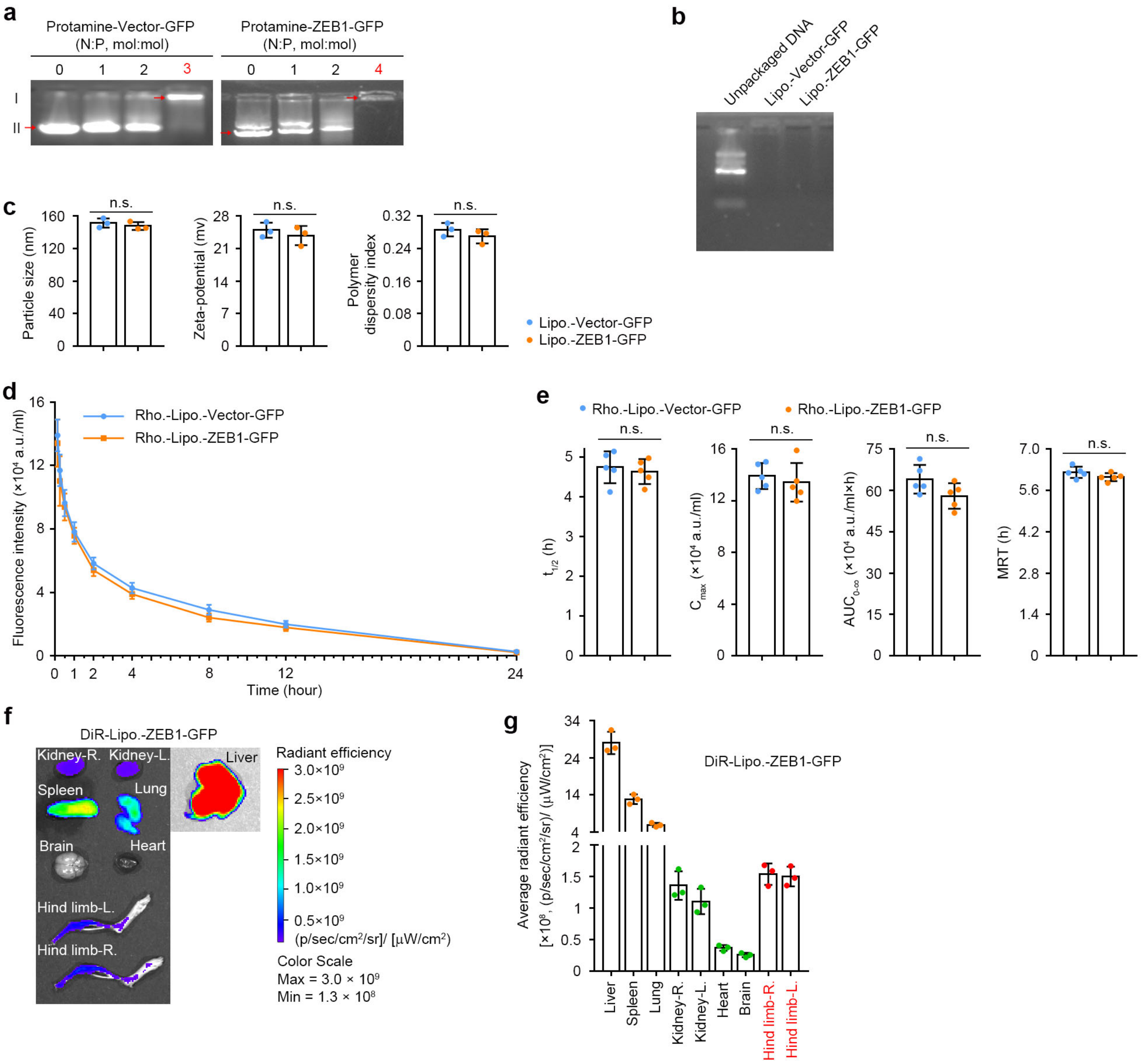

Supplementary Figure 6. Physicochemical characteristics, pharmacokinetics, and biodistribution of DNA-packaged cationic liposomes. a Representative agarose gel electrophoresis images of Protamine-Vector-GFP and Protamine-ZEB1-GFP at different N:P ratios. Images as shown are from three independent experiments. **b** Representative agarose gel electrophoresis images of the final Lipo.-Vector-GFP and Lipo.-ZEB1-GFP. Unpackaged plasmid DNA was used as negative control. Images as shown are from three independent experiments. **c** Particle size, zeta potential, and polymer dispersity index (PDI) of the final Lipo.-Vector-GFP and Lipo.-ZEB1-GFP (n = 3 independent experiments). **d**, **e** Rho.-Lipo.-Vector-GFP and Rho.-Lipo.-ZEB1-GFP were i.v. injected into rats (n = 5, each); The blood samples collected at different times post treatment were centrifuged, and the supernatant plasma was collected for fluorescence intensity measurement. Fluorescence intensity-time curves of DNA-packaged Rho.-liposomes were plotted (**d**), and pharmacokinetic parameters such as $t_{1/2}$ (elimination half-life), C_{max} (maximal plasma concentration), $AUC_{0-\infty}$ (area under the plasma concentration-time curve), and MRT (mean residence time) were calculated using Phoenix WinNonlin 6.4 software (**e**). **f**, **g** Representative ex vivo fluorescence images of hind limbs, kidneys, spleen, lungs, brain, heart, and liver of 8-week-old mice (n = 3) that were i.v. injected with 1.0 mg/kg DiR-Lipo.-ZEB1-GFP and sacrificed 24 h post injection (**f**). DiR fluorescence intensity in organs as shown in **f** were measured (**g**; n = 3 independent experiments). All data are represented as mean ± s.d. n.s., not significant. Differences are tested using unpaired two-tailed Student's *t*-test (**c**, **e**). The source data are provided as a Source Data file.

Supplementary Figure 7

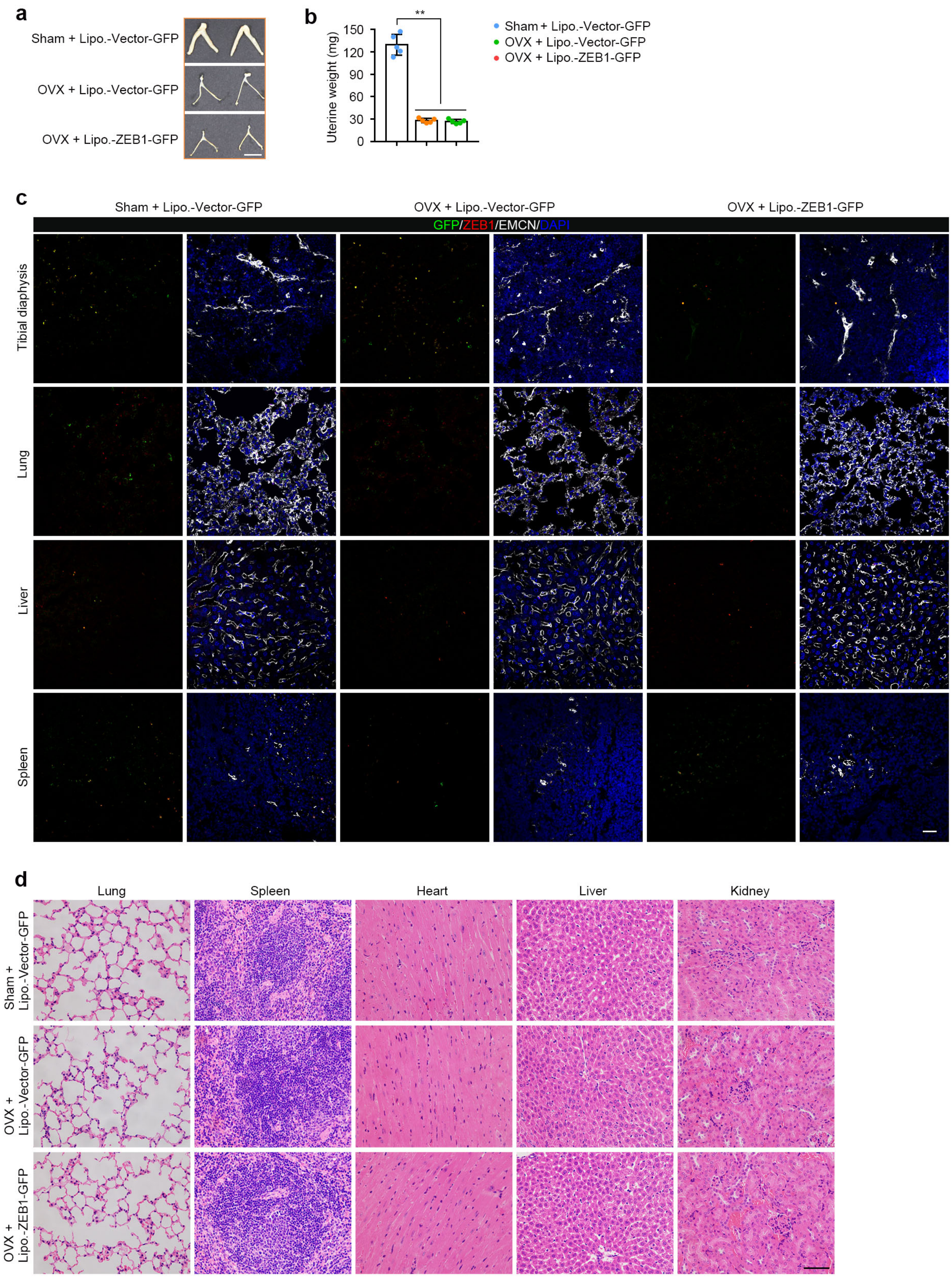

Supplementary Figure 7. *Zeb1*-liposome treatment does not induce histological alterations in the non-skeletal vital organs. a, Gross images of uterus dissected from Sham and OVX mice that were i.v. injected with 4 μg Lipo.-Vector-GFP or Lipo.-ZEB1-GFP for 6 consecutive weeks (designed Sham + Lipo.-Vector-GFP, OVX + Lipo.-Vector-GFP, and OVX + ZEB1-GFP mice, respectively; n = 5, each). Scale bar, 1.0 cm. **b** Comparisons of uterine weights of mice as shown in **a** (n = 5, each). **c** Representative confocal images of GFP/ZEB1/EMCN immunostaining in the diaphysis of tibia and non-skeletal organs (e.g. lung, liver, and spleen) of mice as shown in **a** (n = 5, each). Scale bar, 100 μm. **d** Representative images of H&E-stained non-skeletal organs (e.g. lung, spleen, heart, liver, and kidney) in mice as shown in **a** (n = 5, each). Scale bar, 50 μm. All data are represented as mean ± s.d. ** $P < 0.01$. Differences are tested using one-way ANOVA with Tukey's post hoc test (**b**). The source data are provided as a Source Data file.
